# Hypofractionated Radiotherapy Upregulates Several Immune Checkpoint Molecules in Head and Neck Squamous Cell Carcinoma Cells Independently of the HPV Status While ICOS-L Is Upregulated Only on HPV-Positive Cells

**DOI:** 10.3390/ijms22179114

**Published:** 2021-08-24

**Authors:** Sebastian Wimmer, Lisa Deloch, Michael Hader, Anja Derer, Fridolin Grottker, Thomas Weissmann, Markus Hecht, Antoniu-Oreste Gostian, Rainer Fietkau, Benjamin Frey, Udo S. Gaipl

**Affiliations:** 1 Department of Radiation Oncology, Universitätsklinikum Erlangen, Friedrich-Alexander-Universität Erlangen-Nürnberg (FAU), 91054 Erlangen, Germany; wimmersebastian@gmx.de (S.W.); lisa.deloch@uk-erlangen.de (L.D.); Michael.Hader@uni-bayreuth.de (M.H.); anja.derer@uk-erlangen.de (A.D.); fridolingrottker@web.de (F.G.); thomas.weissmann@uk-erlangen.de (T.W.); markus.hecht@uk-erlangen.de (M.H.); rainer.fietkau@uk-erlangen.de (R.F.); benjamin.frey@uk-erlangen.de (B.F.); 2 Translational Radiobiology, Department of Radiation Oncology, Universitätsklinikum Erlangen, Friedrich-Alexander-Universität Erlangen-Nürnberg (FAU), 91054 Erlangen, Germany; 3 Comprehensive Cancer Center Erlangen-EMN, 91054 Erlangen, Germany; 4 Department of Otorhinolaryngology, Head and Neck Surgery, Friedrich-Alexander-Universität Erlangen-Nürnberg (FAU), 91054 Erlangen, Germany; Antoniu-Oreste.Gostian@uk-erlangen.de

**Keywords:** radiotherapy, immunotherapy, HPV status, HNSCC, immune checkpoint molecules

## Abstract

While the treatment of squamous cell carcinoma of the head and neck (HNSCC) with radiotherapy (RT) is complemented more and more by immunotherapy in clinical trials, little is known about the impact of the human papillomavirus (HPV) status or the applied RT scheme on the immune phenotype of the tumor cells. Therefore, we aimed to examine the impact of the HPV status of four human HNSCC cell lines on cell death and the expression of immune checkpoint molecules (ICMs) after RT with either hypofractionation irradiation (5x3.0Gy) or a high single dose (1x19.3Gy) via multicolor flow cytometry and quantitative PCR at an early time point after therapy. In our study, 5x3.0Gy RT induced high numbers of early and late apoptotic cells independent of the HPV status, but necrosis was only increased in the HPV-positive UM-Scc-47 cells. Generally, the immune stimulatory ICMs (CD70, CD137-L, ICOS-L) were less affected by RT compared to the immune suppressive ones (PD-L1, PD-L2, and the herpesvirus entry mediator (HVEM)). A significant higher surface expression of the analyzed ICMs was found after hypofractionated RT compared to a single high dose; however, regardless of the HPV status, with the exception of ICOS-L. Here, HPV-positive HNSCC tumor cells showed a stronger response to 5x3.0Gy than HPV-negative ones. On the RNA level, only minor alterations of ICMs were observed following RT, with the exception of the HPV negative cell line CAL33 treated with 5x3.0Gy, where PD-L2, HVEM and CD70 were significantly increased. We conclude that the HPV status may not distinctly predict immunological responses following RT, and thus cannot be used as a single predictive marker for therapy responses in HNSCC. In contrast, the patient-specific individual expression of ICMs following RT is preferable for the targeted patient selection for immune therapy directed against distinct ICM.

## 1. Introduction

Squamous cell carcinomas of the head and neck (HNSCCs) are characterized as the sixth most common cancer worldwide [1]. In Germany, they account for a total of 3.8% of all male and 1.8% of all female new cancer cases according to a 2016 survey, while the 5-year survival rate of these tumors is only 47% in men and 63% in women [2]. In addition, patients with locoregional tumor recurrence suffer from severe tumor symptom burden [3]. HNSCCs can be divided into two broad subgroups based on their human papillomavirus (HPV) infection status. HPV-negative HNSCCs are associated with risk factors such as the consumption of carcinogenic substances, i.e., alcohol and tobacco. This also explains the gender-specific prevalence of these tumors, as men have a higher average consumption of these stimulants compared to women [4,5,6]. HPV-positive HNSCCs, on the other hand, are associated with an infection with a high-risk HPV subtype. This latter patient population is characterized by a better survival prognosis compared to the total number of patients and now represents the largest proportion of oropharyngeal carcinomas in many Western countries [6,7,8].

HPV-positive tumors have been shown to be more radiosensitive regarding the clonogenic survival compared to HPV-negative tumors [9]. One of the common features of HNSCCs is the occurrence of tumor hypoxia. This impairs the efficacy of radiotherapy (RT), and it is suggested that the increased radiosensitivity of HPV-positive tumors is partly due to a radiation-induced decrease in the hypoxic fraction [10]. Nevertheless, the improvement of precision RT presumably results in a reduction in side effects rather than in better tumor control in HNSCC. The latter can potentially be optimized by the improvement of simultaneously administered immune checkpoint inhibitors (ICIs) as system therapy [11]. In recent years, clinical studies have been initiated to gain knowledge about immune modulatory properties of the classical therapies for HNSCC, namely RT and chemotherapy (CT) in addition to surgery, and to optimize patient selection for additional immune therapy with ICIs [12,13,14]. For this, knowledge about radiation-induced immune alterations is particularly important, in addition to that on how radiation interacts with the tumor cell DNA.

The immune system plays an important role in both, the development of cancer and the eradication of tumors. In a healthy system, it protects the human body from cancer cells, as these express other proteins due to their altered genome, which are recognized as tumor antigens and trigger an immune response [15]. In order to prevent this recognition and combat by the immune system, tumor cells have developed various mechanisms to evade the immune system [16,17]. An immunosuppressive microenvironment inhibits the invasion and action of immune cells, for example through hypoxia, nutrient deprivation, or immunosuppressive metabolites. Furthermore, tumor cells express inhibitory molecules on their surface, for example the programmed cell death-1 ligand (PD-L1), the ligand for the inhibitory PD-1 receptor on T cells and thus evading a T cell response [18].

It has been demonstrated that RT increases the expression of PD-L1 on tumor cells and thereby inhibits T cell-mediated anti-tumor responses. RT has immune suppressive properties, but also immune stimulatory ones, as, e.g., the induction of the release of danger signals [19]. The latter are predominantly released by necrotic tumor cells, while apoptotic tumor cells have a more immune-suppressive property when being cleared by macrophages [20]. By making use of the immune stimulatory effects of RT with concomitantly inhibiting PD-L1, the efficacy of RT to induce anti-tumor immune responses can efficiently be restored [21].

In a clinical context, the two anti-PD-1 monoclonal antibodies nivolumab and pembrolizumab are approved for the treatment of recurrent HNSCC in the USA since 2016 and in the European Union since 2017 [22]. These drugs can also be used as monotherapy for metastatic or non-resectable HNSCC. Even though a small proportion of the HNSCC patients do respond very well to inhibition of the PD-1/PD-L1/-L2 axis alone and in multimodal settings, still the vast amount does not.

In order to identify/select patients who are likely to respond positively to therapy with immune checkpoint inhibitors, research has moved specifically towards prognostic biomarkers in recent years [23]. We just recently identified a liquid immune profile-based signature to predict response of patients with recurrent/metastatic solid cancer to immune checkpoint inhibitors [24]. Furthermore, the limited response could be partly due to additional upregulation of other immune-suppressive immune checkpoint molecules on HNSCCs, as has already been demonstrated for the herpesvirus entry mediator (HVEM) in breast cancer [25]. However, the overall knowledge about the expression of key immune checkpoint molecules in addition to PD-L1 in HNSCCs following RT is scarce [26], and thus is in the focus of our here presented examinations. Additionally, agonists targeting the inducible T cell co-stimulator (ICOS, CD278) are currently under clinical investigation also for HNSCC, with the aim to, synergistically with anti-PD-1, inhibit the suppressive activity of regulatory T cells and to potentiate the anti-tumor activity of effector T cells [27]. Further, stimulation of T cells by binding of CD137-L to CD-137 on activated T cells is another approach to boost anti-tumor immune responses. It has already been demonstrated that HPV-positive HNSCC tumor clearance is increased by a combination of RT with CT and CD137 ligation [28]. As CAR T cells are currently under investigation to target CD70 on HNSCC [29], we also investigated the expression of CD70 on HNSCCs following radiation exposure.

One characteristic biomarker that often applies to a large proportion of HNSCC patients is an HPV infection. HPV infections, the most common sexually transmitted disease in the United States, heal successfully in the vast majority of infected individuals [30]. In the event of failure of the immune response or infection with one of the fifteen high-risk HPV types, HPV may persist in the body, which in turn increases the risk of tumor formation. Especially high-risk HPV types that persist for years increase the likelihood of incorporation of parts of the viral genome into the genetic material of the epithelial cell [31]. The integration of the viral genome can lead to overexpression of the oncoproteins E6 and E7, which can ultimately lead to malignant degeneration of the infected epithelial cells, since these two oncoproteins influence growth-regulating intracellular signaling pathways and inactivate tumor suppressor proteins, among others [32,33]. HPV-positive HNSCCs are discussed to have a higher immunogenicity [34]. They are characterized by a higher CD8+ T cell infiltration [12,35]. However, whether differences in the expression of immune checkpoint molecules do exist between HPV-positive and HPV-negative HNSCC is still not known, and is therefore in the focus of our here presented work.

Regarding the influence of the HPV status, accelerated RT fractionation schemes, aiming towards achieving a reduced proliferation capability of tumor cells, and thus reducing the likelihood of a relapse, have also been under investigation for several years [36,37]. In recent years, as more and more inhabitants of middle- to low-income countries have been shown to suffer from tumors of the head and neck area, calculations predict that 50% of patients will not be able to gain timely access to therapies while additional resource constraints apply, and thus altered fractionation schemes have been moved even more into the focus of researchers and clinicians. Therefore, an increasing number of studies evaluate hypofractionated-accelerated fractionation schemes also for HNSCC patients [38]. However, as these forms of treatment stretch the tolerance of normal tissue responses, it is important to closely look into these limitations. In this matter, there are an increasing number of studies, e.g., the multicentric trial of hypo-vs normo-fractionated RT in HNSCC, HYPNO (NCT0765503) study, applying 55Gy in 20 fractions and 4 weeks, addressing these questions. So far, no significant disadvantages in terms of local control, overall survival and quality of life have been found [38,39], and there is evidence that intensifying the treatment by either altering RT fractions or adding concurrent therapies might even improve the outcome in patients [38]. The optimal radiation dose and fractionation for combination of RT with ICM still has to be identified [40]. In order to do so, knowledge about early alterations of the immune phenotype of HNSCC tumor cells following RT with a distinct fractionation approach is mandatory.

To sum up, HPV-associated HNSCC tumors generally respond better to anticancer treatments. It is assumed that this is due to a better response to RT, but data about the role of the immune phenotype of the tumor cells in this setting are scarce [16]. We therefore hypothesize that the immune phenotype of head and neck cancer tumor cells is changed by the treatment with RT depending of the fractionation approach and differs in HPV-positive compared to HPV-negative tumors. Therefore, we analyzed the response of HPV-negative and HPV-positive HNSCC cell lines to different RT regimens (hypofractionated and high single dose) with regard to cell death induction as well as the expression of immune checkpoint molecules. Since concurrent application of RT with ICIs seems to be the most beneficial, we here focused on early changes following radiation exposure [41].

## 2. Results

### 2.1. 5x3.0Gy Induces High Numbers of Early and Late Apoptotic Cells Independent of the HPV Status, While Necrosis Is Only Induced in UM-Scc-47 Cells

**Figure 1:**
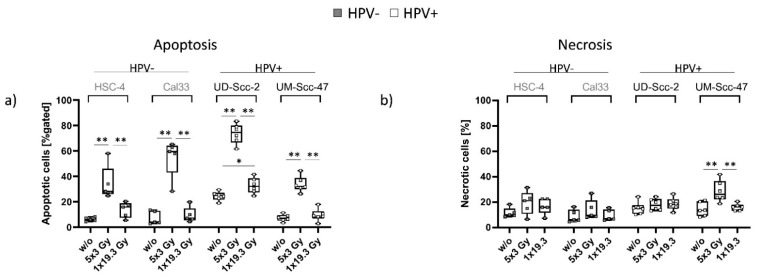
Cell death forms on day 7 for the two HPV-negative cell lines (HSC-4 and CAL33; grey dots) and the two HPV-positive cell lines (UD-Scc-2 and UM-Scc-47; white dots). The graphs show the percentage of early and late apoptotic (**a**) and necrotic cells (**b**) in the total cell count for all three treatment conditions, untreated (*w*/*o*), 5x3.0Gy and 1x19.3Gy n_hpv−_ = 5; n_hpv+_ = 6 biological independent experiments. Graphs show median + min to max, and a two-sided Mann–Whitney U test was performed for comparisons of treatments within one cell line: * *p* < 0.05, ** *p* < 0.01.

A significant increase in (early and late) apoptosis independently of the HPV status, but dependent on the fractionation scheme, was observed. In all examined cell lines, hypofractionated RT(5x3.0Gy) resulted in a significant increase in apoptotic cells in comparison to untreated and 1x19.3Gy irradiated cells. The HPV-positive cell line UD-Scc-2 however, not only showed the highest rate of apoptotic cells, but also a significant increase in apoptotic cells after 1x19.3 in comparison to untreated controls. Primary necrosis () remained stable across all treatments, with the exception of HPV-positive UM-Scc-47, where a significant increase in necrotic cell numbers was found after 5x3.0Gy in comparison to untreated controls and 1x19.3Gy single dose RT (Figure 1b).

### 2.2. The Applied RT Fractionation Scheme Rather Than the HPV Status Affects the Surface Expression of Immune Checkpoint Molecules on HNSCC Tumor Cells, with the Exception of ICOS-L

**Figure 2:**
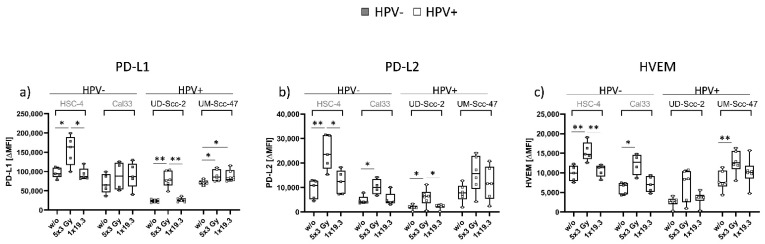
Cell surface expression of immune suppressive checkpoint molecules PD-L1 (**a**), PD-L2 (**b**), and HVEM (**c**) on the cell surface of HPV-negative (grey dots) and HPV-positive (white dots) HNSCC cell lines with n_hpv−_ = 5; n_hpv+_ = 6 biological independent experiments. Cells were subjected to either a hypofractionated irradiation regimen (5x3.0Gy) or a single high dose of 19.3Gy. ΔMFI was calculated by subtracting the respective stained samples from Zombie-only-treated samples. Graphs show median + min to max, and a two-sided Mann–Whitney U test was performed for comparisons of treatments within one cell line: * *p* < 0.05, ** *p* < 0.01.

We then investigated the expression of various immune suppressive (PD-L1, PD-L2, HVEM) checkpoint molecules on the cell surface:

As shown in Figure 2, regulation of immune suppressive checkpoint molecules was independent of the HPV status of the respective cell lines, but rather dependent on the applied fractionation scheme. The expression of PD-L1 was significantly increased in HSC-4, UD-Scc-2, and UM-Scc-47 cells after 5x3.0Gy, while UM-Scc-47 additionally showed a significant increase after 1x19.3Gy. PD-L2 was found to be significantly increased in HSC-4, Cal-33, and UD-Scc-2 cells, while UM-Scc-47 showed a slight increase after 5x3.0Gy and 1x19.3Gy (Figure 2b). HVEM was also found to be increased after 5x3.0Gy, with statistical significance found in HSC-4, Cal-33, and UM-Scc-47 cells (Figure 2c).

**Figure 3:**
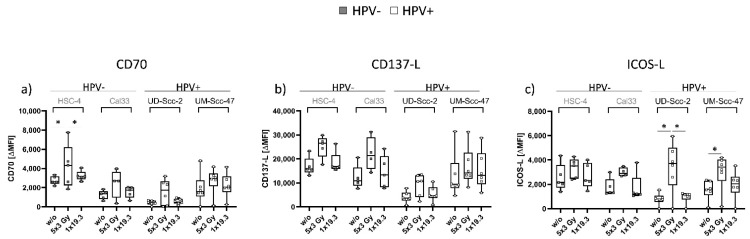
Cell surface expression of immune stimulatory checkpoint molecules CD70 (**a**), CD137-L (**b**) and ICOS-L (**c**) on the cell surface of HPV-negative (grey dots) and HPV-positive (white dots) HNSCC cell lines with n_hpv−_ = 5; n_hpv+_ = 6 biological independent experiments. Cells were subjected to either a hypofractionated irradiation regimen (5x3.0Gy) or a single high dose of 19.3Gy. ΔMFI was calculated by subtracting the respective stained samples from Zombie-only-stained samples. Graphs show Median + min to max and a two-sided Mann–Whitney U test was performed for comparisons of treatments within one cell line: * *p* < 0.05.

The immune stimulatory ICMs were less affected by RT compared to the immune suppressive ones (Figure 2). CD70 was significantly increased in HSC-4 cells after 5x3Gy, while Cal33 and UD-Scc-2 only showed a tendency of increased expression following RT with 5x3.0Gy. In UM-Scc-47, CD70 expression remained stable throughout all treatment regimens (Figure 3a). No significant impact of RT was found for the expression of CD137-L; however, similar to CD70, HSC-4, Cal33, and UD-Scc-2 showed a slight upregulation after 5x3.0Gy. Again, in UM-Scc-47 cells, no clear alteration of CD137-L expression on the cell surface was found, independently of the fractionation scheme (Figure 3b). While no significant ICOS-L alterations were found in HPV cell lines, HPV+ UD-Scc-2 and UM-Scc-47 showed a significant upregulation of ICOS-L on their cell surface after 5x3Gy, in comparison to untreated controls (Figure 3c).

### 2.3. A Dose of 5x3.0Gy Slightly Alters the Gene Expression of ICMs in Cal33 and UD-Scc-2 Cells

**Figure 4:**
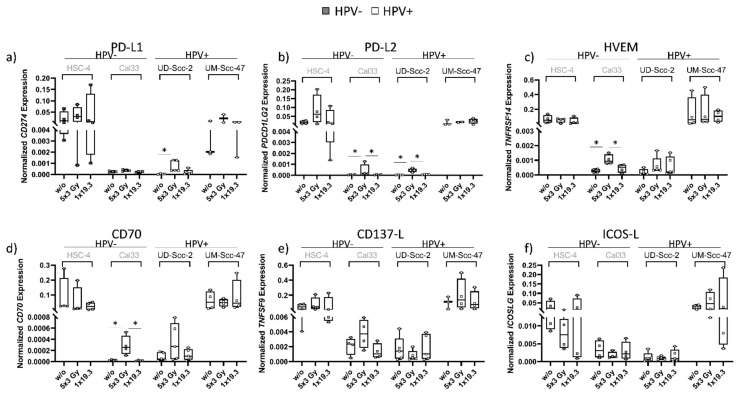
Normalized gene expression of immune suppressive (PD-L1 (**a**), PD-L2 (**b**), HVEM (**c**) and immune stimulatory (CD70 (**d**), CD137-L (**e**), ICOS-L (**f**)) checkpoint molecules of HPV-negative (grey dots) and HPV-positive (white dots) HNSCC cell lines. Cells were subjected to either a hypofractionated irradiation regimen (5x3.0Gy) or a single high dose of 19.3Gy. Normalized gene expression was calculated by normalizing samples to the following housekeeping genes: HPV−: ACTB, RPL27, RPL30, RPS18; HPV+: ACTB, RPL27, RPL30, RPS18, UBC. Graphs show Median + min to max for n_HSC-4_ = 4, n_Cal33_ = 5, n_UD-Scc-2_ = 5, n_UM-Scc-47_ = 3 biological independent experiments and a two-sided Mann–Whitney U test was performed for comparisons of treatments within one cell line: * *p* < 0.05.

Figure 4 shows gene expression analysis of ICMs carried out via qPCR. In general, the expression of genes of interest was found to be rather heterogenic and was dependent rather on the cell line itself as well as the fractionation scheme than on HPV status. HSC-4 and UM-Scc-47 cells had a generally higher gene expression level of ICMs than Cal33 and UD-Scc-2, but radiation-dependent significant modulations of ICMs were only observed in Cal33 and UD-Scc-2. Similar to surface expression, UD-Scc-2 cells showed a significant increase in PD-L1 levels at 5x3.0Gy in comparison to untreated controls, while the other cell lines did not. PD-L2 levels were found to be significantly elevated at 5x3.0Gy in Cal33 and UD-Scc-2 cells, while the same was only seen in Cal33 cells for HVEM and CD70 gene expression, where again, 5x3.0Gy resulted in significantly elevated expression levels.

## 3. Discussion

### 3.1. Hypofractionated RT Has a Bigger Influence on Cell Death than HPV Status in the Examined Four HNSCC Tumor Cell Lines

In the clinic, HPV-positive HNSCC tumors are generally associated with a better prognosis, and thus improved therapy outcome [42]. They often show a better response to RT and are therefore considered more radiosensitive than HPV-negative HNSCCs [10,43,44]. While numerous studies have demonstrated a higher radiosensitivity for HPV-positive tumors [45], Rieckmann et al. found HPV-positive tumors to be a very heterogeneous group, that, depending on the examined cell line, often show overlapping results with HPV-negative tumors [46].

Our studies regarding the cell death of HNSCC cells after hypofractionated (5x3.0Gy) or single high dose (1x19.3Gy) RT showed that the applied RT scheme has a bigger impact on cell death than the HPV status in the four cell lines we have looked at (Figure 1). Hypofractionated RT treatment significantly induced apoptosis in all four cell lines, while 1x19.3 showed only minimal impact on cell death, with the exception of UD-Scc-2 cells, where a significant increase also after 1x19.3Gy was observed. When taking a closer look, the HPV-positive UD-Scc-2 had the overall highest rate of apoptotic cells in comparison to all examined cell lines, while HPV-positive UM-Scc-47 had the lowest rates of apoptotic cells. However, in contrast to all other examined cell lines, UM-Scc-47 alone showed a significant increase in necrotic cell numbers, also at 5x3.0Gy, while 1x19.3Gy had no significant impact on necrosis at the examined early time point following radiation exposure.

As apoptosis is generally considered to be a rather immunosuppressive and necrosis is considered to be an immune-activating form of cell death, necrosis is therefore the preferred outcome in cancer therapy, as it is expected to induce a better anti-tumor response in treated patients [16,47]. When looking at cell death forms in our experimental setting, UM-Scc-47 therefore seems to possess the most immunogenic potential. We thus conclude that, while RT itself had the biggest impact on cell death, HPV-positive cell lines showed slightly better responses to RT than HPV-negative ones. This finding complies with the general understanding of HPV-positive HNSCC tumors being more susceptible to RT than HPV-negative ones [48]. Recent data further indicate that HPV-positive tumors have a better response to immune therapies [49]. This suggests that HPV-positive and HPV-negative tumor cells have different immune phenotypes. HPV-positive oropharyngeal tumors are characterized by a beneficial immune micro-environment with high levels of tumor-infiltrating lymphocytes [50]. Increased levels of both circulating and tumor infiltrating CD8+ T cells are related to better survival in HPV-positive HNSCC [51]. However, these cytotoxic T cells could be suppressed by immune checkpoint molecules that might change their expression following RT.

### 3.2. A Dose of 5x3.0Gy Mainly Increases the Expression of Immune Suppressive ICMs Independently of the HPV Status

As mentioned above, ICMs are key regulators of T cell-mediated anti-tumor immune responses. However, a large number of patients do not respond properly to immune therapies targeting such ICMs. Therefore, improving existing immunotherapies with optimized RT schemes and identifying other RT-regulated ICMs besides PD-L1 is an important step to increase response rates to radio-immunotherapies [52,53].

The integration of the HPV genome into the cellular genome of the host can lead to an increased expression of the two oncoproteins E6 and E7 [7] and these mutations can then lead to a change in the immunological microenvironment [54,55]. However, whether this affects the expression of ICMs in cancer cells and thereby the immune phenotype as well as the impact of various forms of RT on it is not known. Consequently, we treated HPV-negative and -positive HNSCC cell lines with two clinically relevant RT schemes and subsequently checked for surface and gene expression of several ICMs being of potential importance in HNSCC as outlined in the introduction.

We found that, in general, irradiation with 5x3.0Gy led to an increase in the surface expression of the immune inhibitory checkpoint molecules PD-L1, PD-L2 and HVEM (Figure 2). A significant increase was found on the surface of HSC-4 cells for all of the mentioned ICMs after 5x3.0Gy, while 1x19.3Gy remained stable and comparable to untreated levels. Likewise, no effect was observed on mRNA level at the same timepoint for either treatment (Figure 4), suggesting that the upregulation of inhibitory ICIs seems to be regulated on a protein level (Figure 2 and Figure 3). For HPV-negative Cal33 tumor cells, an upregulation on the cell surface of PD-L2 and HVEM after 5x3.0Gy was found (Figure 2b,c). qPCR analysis showed that PD-L2 and HVEM gene expression is still significantly upregulated at the same time, possibly suggesting a long-lasting response on the surface (Figure 4b). These data suggest that targeting PD-L2 and HVEM should be taken into consideration in individual HNSCC patients that receive RT. While PD-L2 is already targeted when the receptor PD-1 is inhibited, novel immune checkpoint inhibitors against HVEM should be tested in the future also for HNSCC, as in melanoma it was found that HVEM has a broader expression than even PD-L1 and serves as a negative prognostic marker [56].

Irradiation of HPV-positive UD-Scc-2 with 5x3Gy resulted in a significant upregulation of PD-L1 and PD-L2 (Figure 2a,b), while mRNA levels of PD-L1 and PD-L2 (Figure 4a,b) were significantly enhanced at the same timepoint, again suggesting a long-lasting upregulation of these ICMs on the surface. UM-Scc-47 showed a significant increase in the surface expression of PD-L1 and HVEM 24 h after the last RT (Figure 2a,c), mRNA levels from samples taken at the same timepoint, however, showed no differences between RT treatments and control (Figure 4). Interestingly, gene expression levels of HPV-negative HSC-4 and HPV-positive UM-Scc-47 were similar and much higher for all examined molecules than the gene expression levels of Cal33 (HPV-negative) and UD-Scc-2 (HPV-positive), which were also found to be expressed on a similar level (Figure 4a–c).

We thus conclude that while individual tumor cell lines show individual responses in terms of intensity and duration of ICM expression, RT with 5x3.0Gy has an overall stimulatory effect regarding the expression of suppressive ICMs, independent of cell line and HPV status. While PD-L1 expression is generally enhanced in HPV-positive HNSCCs [57], it is not HPV-specifically regulated following radiation exposure. These results will have to be confirmed with more HNSCC cell lines, which need, however, to first be generated, since the amount of available HNSCC cell lines for research is still limited. Furthermore, the dynamics of the responses is of importance [58]. We, and others, have previously shown that the expression of immune checkpoint molecules and other immune modulations in tumor cells following radiation exposure start as early as 12 h after RT and peak at 24–48 h [59,60]. Afterwards, the expression either remains stable or is normalized. Since concurrent application of RT with immune checkpoint inhibitors seems to be the most beneficial [41] and since immune cells infiltrate into tumors as early as 1 day following radiation exposure [61], we here also focused on early changes following radiation exposure. Combining RT with IT with distinct antibodies targeting ICMs in a multimodal setting at the right time and in combination with the optimal dose thus might not only aide in achieving a local but also systemic tumor control by overcoming tumor-induced immunosuppression [62,63].

### 3.3. ICOS-L Shows Significantly Enhanced Surface Expression after 5x3.0Gy Only in HPV-Positive HNSCC Cell Lines

When looking specifically at immune stimulatory checkpoint molecules, 5x3.0Gy again had the overall highest impact on regulation of checkpoint molecules while 1x19.3Gy resulted in expression levels that were close to those of controls (Figure 3). Similar to immune suppressive checkpoint molecules, HSC4 and UM-Scc-47 again showed higher gene expression levels than Cal33 and UD-Scc-2 in qPCR analysis (Figure 4).

For CD70 surface expression (Figure 3a), we found a significant increase after 5x3.0Gy in HSC-4 tumor cells; however, this effect was not visible on mRNA level (Figure 4d). Furthermore, Cal33 and UD-Scc-2 showed a tendency of increased surface expression of CD70. A similar tendency was visible in gene expression levels, whereas CD70 expression in Cal33 was significantly enhanced after 5x3.0Gy (Figure 4d), again suggesting a possible surface upregulation at a later timepoint. UM-Scc-47 showed no influence of RT on expression levels, either on the surface or on mRNA level at the observed time point. For CD137-L (Figure 3b and Figure 4e), similar tendencies were observed after 5x3.0Gy, but without any significant alterations. In both checkpoint molecules, UM-Scc-47 reacted differently than the other three cell lines, as no impact of RT on either the surface or mRNA level could be observed. This suggests the observed effects to be rather due to the individual cell lines rather than an influence of the HPV status.

However, when looking at ICOS-L, only the HPV-positive cell lines were characterized by a significantly increased ICOS-L surface expression after RT with 5x3.0Gy levels (Figure 3). As ICOS-L is a co-stimulatory ligand that is involved in optimizing T cell function, one could speculate that RT-induced increased expression might contribute to a better prognosis and responsiveness to RT of HPV-positive HNSCCs [27,48].

The cumulative work of the last few years suggests that HPV-positive HNSCC tumors have a better prognosis and responsiveness to radiotherapy treatment. In addition, HPV-positive HNSCCs are also often described to be more homogenous in their behavior than HPV-negative ones [32,43,45,48]. We here found the individual responses of the HNSCC cell lines as well as the influence of RT schemes to be more pronounced than HPV status in most of the markers for the immunogenic properties of the tumor cells we looked into in the four cell lines our study was limited to. Nevertheless, a patient-specific approach concerning HPV status might be the most promising approach in the clinic.

Notably, a hypofractionated RT scheme of 5x3.0Gy was very effective in terms of inducing cell death and modulating immune checkpoint molecules on the surface of HNSCC cells, and partly also on a mRNA level. This should be considered when designing new multimodal tumor therapies with adapted RT protocols [40]. However, as current studies are carried out with rather small patient cohorts and little is known about long-term effects, especially with regard to normal tissue tolerance [38], additional preclinical research in this area is needed.

Taken together, our findings suggest a holistic approach to be the most promising one for optimizing multimodal treatments for HNSCC instead of particularly focusing on HPV status, one RT fractionation scheme, or single ICM inhibition. For a large cohort of patients, examining immune matrices in individual tumors depending on distinct RT protocols might add additional value in the future.

## 4. Materials and Methods

### 4.1. Cell Lines and Cultivation

Four different human cancer cell lines from head and neck tumors (HSC-4: tongue squamous cell carcinoma; epithelioid; Cal33: tongue squamous cell carcinoma; UM-Scc-4: tongue squamous cell carcinoma; UD-Scc-2: hypopharyngeal squamous cell carcinoma) were used, each kindly provided by Dr. T. Rieckmann (Hamburg, Germany). The two cell lines UM-Scc-47 and UD-Scc-2 are HPV-positive, whereas the cell lines HSC-4 and CAL33 are HPV-negative ones. All cell lines were cultured in modified D10 medium (DMEM; PAN-Biotech GmbH, Aidenbach, Germany) supplemented with 10% fetal bovine serum (Biochrome AG, Berlin, Germany) and 100 U/mL of Pen/Strep (Gibco, Carlsbad, CA, USA) under standard culture conditions (37 °C, 5% CO_2_ and 95% humidity).

### 4.2. Treatments and Sampling

**Figure 5:**
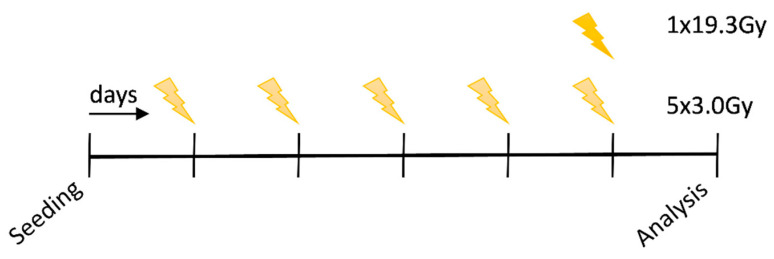
Irradiation schedule of the HNSCC cell lines with 5x3.0Gy (hypofractionated) and 1x19.3Gy (high single dose). Seeding of the cells was carried out on day 1, irradiation with 5x3.0Gy on day 2–6 and 1x19.9Gy on day 6, respectively. On day 7, analysis of the cells via flow cytometry or collection of cell pellets in TriFast was performed. In the above scheme, each vertical line represents one day of the experiment.

For the experiments, the cell lines were seeded and cultivated under standard conditions (37 °C, 5% CO_2_ and 95% humidity). After they had grown for 24 h, the respective cell culture flasks were exposed to irradiation using a hypofractionated scheme with a single dose per fraction of 3.0Gy of X-rays (120 kV, 12.2 mA for 0.5 min) for 5 days and a scheme with a single dose of 19.3Gy (120 kV, 12.2 mA for 0.5 min) on d6, using a Isovolt Titan series-GE Technologies (Hürth; Germany) X-ray machine. Non-irradiated controls were seeded in parallel for each scheme. On experimental day 7, an evaluation of all conditions, i.e., treatment and control, was performed using a CytoFLEX S flow cytometer (Beckman Coulter, Brea, CA, USA) and samples were collected for subsequent RNA analyses. The complete procedure of the experimental set-up is shown in Figure 5.

### 4.3. Cell Death Measurement Using AnnexinV/Propidium Iodide Staining Solution

**Figure A1:**
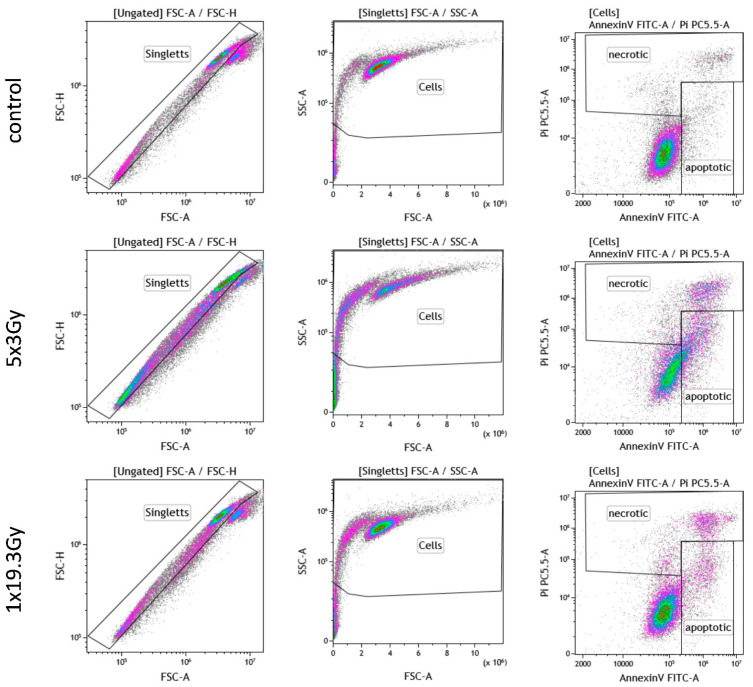
Gating strategy for cell death measurement with AnnexinV/Propidium iodide staining. **Left**: Forward-side-scatter to separate single cells from cell clusters. **Middle**: Separation of cells from cell debris. **Right**: Separation of the different cell death forms with AnnexinV-FITC and Propidium iodide. Primary necrotic cells: Ax+/Pi++, early apoptotic cells: Ax+/Pi−, late apoptotic cells: Ax+/Pi+. Shown is the gating strategy using an exemplary staining for HSC-4 cells that were evaluated on day 7 of the experiment.

On day 7 of the experiment, multicolor flow cytometry was used to analyse the different forms of cell death. For this purpose, tumor cells were harvested with trypsin and 1 × 10^5^ cells per 96-well were incubated with AnnexinV (AxV)/propidium iodide (PI) staining solution (1.0 µg/mL of PI and 0.5 µg/mL of FITC-labeled AxV). Subsequent analysis was performed using a CytoFLEX S flow cytometer. The gating strategy is shown in Appendix A Figure A1. Cells being positive for both AxV and PI were defined as necrotic ones, with those being positive for AxV and negative for PI being defined as early and late apoptotic ones.

### 4.4. Detection of Immune Checkpoint Molecule Expression by Multicolor Flow Cytometry

**Figure A2:**
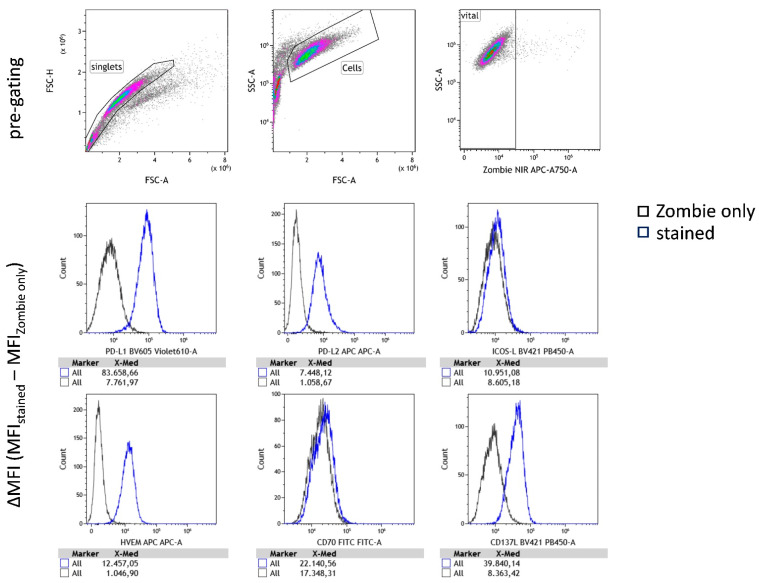
Exemplary gating strategy for analyses of cell surface expression of immune checkpoint molecules via multicolor flow cytometry. Pre-gating: identification of single cells (“singlets”), differentiation of cells from debris (“cells”) and vital cells via Zombie live/dead stain (“vital”). X-Med of various antibodies for immune checkpoint molecules was taken and X-Med values of Zombie only stained samples was subtracted for ΔMFI calculation.

The expression of immune checkpoint molecules on the surface of the tumor cells was analyzed on day 7. For this purpose, the harvested tumor cells were incubated with the antibodies listed in Table 1. Subsequent analysis was performed using a CytoFLEX S flow cytometer and two staining panels for checkpoint expression analysis. The mean fluorescence intensity (MFI) of the respective immune checkpoint ligands in the sample stained with the antibody mix is corrected to the MFI of the control, which was stained with Zombie NIR only, for its background and presented as ∆MFI in the graphs. The Zombie NIR stain was used to assess live vs. dead status of the HNSCC cells in the measurements of the expression of immune checkpoint molecules. This dye is non-permeant in live cells, but permeant in the cells with disturbed membranes. The gating strategy can be found in Appendix B Figure A2.

### 4.5. Quantitative PCR Analyses

Isolation of RNA was done via phenol/chloroform extraction in TriFast (peqlab, Darmstadt, Germany) according to the manufacturer’s protocol and purity of RNA was determined using a P330 nanophotometer (Implen, Munich, Germany). Pre-made Prime PCR primers from Bio-Rad (Hercules, CA, USA; see Table 2) were used to perform the qPCR reactions. qPCR analysis was performed using the DyNAmo ColorFlash SYBR Green qPCR Kit (Thermo Fisher, Waltham, MA, USA). In addition, a separate non-reverse transcription control was carried out for each sample and a non-template control was carried along for each primer. Results were analyzed using Bio-Rad CFX Manager software. For each cell line, multiple housekeeping genes were used for normalization (HPV-negative cell lines: ACTB, RPL27, RPL30, RPS18; HPV-positive cell lines: ACTB, RPL27, RPL30, RPS18, and UBC). Data are shown as normalized data as calculated by the Bio-Rad CFX manager 3.1. The used primers are listed in Table 2.

### 4.6. Statistical Analysis

Data are shown as median + min to max. Statistical analysis was performed using GraphPad Prism V8.3.0 software (Graph Pad Software, LLC; San Diego, CA, USA). For all data, a two-sided Mann–Whitney U test was performed for comparisons of treatments within one cell line. Significances were considered as follows: * *p* < 0.05, ** *p* < 0.01, and *** *p* < 0.001.

## Figures and Tables

**Table 1:** List of antibodies used to analyse the surface expression of the immune checkpoint molecules by multicolor flow cytometry.

Marker	Fluorochrome	Manufacturer	Cat. Nr.
PD-L1 (CD 274)	BV 605	BioLegend	329,724
PD-L2 (CD 273)	APC	BioLegend	345,508
ICOS-L (CD 275)	BV 421	BD Bioscience	564,276
HVEM (CD 270)	APC	BioLegend	318,808
TNFRSF9 (CD13L)	BV 421	BioLegend	311,508
CD27-L (CD70)	FITC	BioLegend	355,106
Zombie NIR	APC-A750	BioLegend	423,105

**Table 2:** List of primers of genes of interest and reference genes used for qPCR analysis.

Gene Symbol	Gene Name	Bio-Rad Unique Assay ID
Genes of Interest		
CD274	CD274 molecule (PD-L1)	qHsaCID0036468
Pdcd1lg2	programmed cell death 1 ligand 2 (PD-L2)	qHsaCID0015625
CD70	CD70 molecule (CD70)	qHsaCID0038688
TNFRSF14	tumor necrosis factor receptor superfamily, member 14 (HVEM)	qHsaCID0013930
TNFSF9	tumor necrosis factor (ligand) superfamily, member 9 (CD137-L)	qHsaCED0003427
ICOS-L	inducible T-cell co-stimulator ligand	qHsaCED004588
Reference Genes		
RPS18	ribosomal protein S18	qHsaCED0037454
RPL30	ribosomal protein L30	qHsaCED0038096
RPL27	ribosomal protein L27	qHsaCED0044386
ACTB	actin, beta	qHsaCED0036269
UCB	ubiquitin C	qHsaCED0023867

## Data Availability

The data presented in this study are available on reasonable request from the corresponding author.
